# Changing the frame: the impact of welfare narrative presentation on citizen choices about cow–calf separation

**DOI:** 10.3389/fvets.2026.1813343

**Published:** 2026-09-14

**Authors:** Abbie-Louise Smith, Charlotte Doidge, Jasmeet Kaler

**Affiliations:** Ruminant Population Health Group, School of Veterinary Medicine and Science, University of Nottingham, Nottingham, United Kingdom

**Keywords:** calf welfare, cow–calf separation, dairy, factorial survey, multilevel modelling

## Abstract

Cow–calf separation, a highly controversial practice of the dairy industry, is a frequent topic of criticism amongst citizens. The divergence in opinion that exists between the public and farmers means that the continued occurrence of this practice on dairy farms results in friction in the producer-consumer relationship. Although research into the public's perceptions of this practice is emerging, investigations into how these change using different experimental methods are lacking. This study aimed to understand how welfare message framing influences UK citizens' decision-making on cow–calf separation, using a factorial survey approach. Responses were received from 503 participants. Four of the five welfare domains, *physical environment, health, behavioural interactions*, and *mental state*, significantly influenced respondents' decision-making, with a positive welfare outcome in each domain increasing respondents' agreement with cow–calf separation and resulting in a more positive rating of the welfare impact on the calf. Respondent variables that had a significant influence on respondents' ratings were age, level of knowledge about the dairy industry, dietary preference, presence of links to the dairy industry, and type of area lived in. The pre-survey level of agreement with the practice also significantly influenced respondents' ratings. A clear understanding of citizens' viewpoints is critical in maintaining the dairy industry's social license to operate; therefore, insights gained from this study could provide a starting point for collaborative communication between the public and the dairy industry stakeholders to bridge awareness gaps on this practice, allowing for better industry transparency and improved mutual understanding of the various viewpoints held.

## 1 Introduction

Separation of the calf from the cow within 24 h of birth is a highly contentious and frequently criticised practice of the dairy industry due to its poor alignment with the process of calf rearing in a natural setting (1) as well as the negative welfare implications it poses on both the cow and the calf (2). The public are often a prominent voice of opposition against the practice, a stance that is echoed globally (3–5). As a consequence, its continued occurrence on most dairy farms acts as a trigger for friction in the producer-consumer relationship.

In contrast to the general public, farmers and veterinarians are considered advocates of early cow–calf separation (1) and do not consider it a prominent welfare issue affecting dairy calves (6). Some of the reasons cited for separating calves shortly after birth include ensuring the timely administration of a sufficient quantity of colostrum (7), minimising disease transmission to the calf (8), and not having the space allowance to provide prolonged cow–calf contact (9). It is also believed that separating calves earlier helps to minimise the emotional distress that is felt by both the cow and the calf (10–12). However, there is limited evidence to support a health benefit of early separation (13). Additionally, later separation of the calf has been found to improve growth rates (14), allow for the development of a normal social repertoire (10) and minimise pessimistic tendencies (15), and reduce the occurrence of diseases such as mastitis in the cow (13). The presence of conflicting evidence which highlights pros and cons to both early and late separation indicates that there is still a lack of consensus, both within the livestock industry and the scientific literature, on the optimal time to separate calves.

Divergence in opinion on cow–calf separation is most pronounced amongst members of the public, who tend to be critical of the practice and its continued occurrence on dairy farms. The public's perception of cow–calf separation has received attention in the literature, with the opinions of German citizens (3), Brazilian citizens (4), and American and Canadian citizens (1, 5) being investigated. However, to date, there have been no studies investigating the perceptions of UK citizens on cow–calf separation. Cross-country studies on citizens' perceptions of agricultural practices are important as individuals' beliefs are shaped by social norms, cultural factors, and societal values which inherently vary between countries (16); therefore, it cannot be assumed that the perceptions of citizens in one country match the perceptions of another. Across these studies, rejection of the practice by the participants ranged from 44% (1) to 69.2% (4). The most commonly cited reason for opposing cow–calf separation amongst the public was ‘unnaturalness' (1, 3, 17), which is unsurprising as the public's perception of welfare is heavily centred around natural practices (18). Additionally, the tendency of the public to anthropomorphise agricultural contexts often means that the cow–calf bond is likened to the bond shared by a mother and child (19). Other reasons that are commonly shared for opposing the practice include potential harms to the health of both the calf and cow (20), negative impacts on the cow and calf's emotions (4) and separation distress (1). Although citizens are strongly vocal about their rejection of this practice on dairy farms, many studies have revealed that a large proportion of participants were unaware of cow–calf separation (1, 4, 20). Bridging this awareness gap through industry transparency and the dissemination of accurate information will assist citizens in making informed decisions on their views of the practice.

Although citizens' perceptions of cow–calf separation have been investigated in some countries, intervention studies that use different techniques to investigate how the public's perception of cow–calf separation changes in different contexts are limited. Hotzel et al. (4) is one example of such intervention study, where the quantity of information provision and its influence on the public's acceptability of the practice was investigated. The study found that providing citizens with additional information on the practice and its production and welfare implications did not increase the public's acceptance of the practice. Similarly, Ventura et al. (21) investigated citizens' perceptions of management practices prior to and after a visit to a working dairy farm. It was identified that whilst the participants' concerns for some management practices were mitigated by the visit, their opinion on cow–calf separation remained unchanged. To date, no such studies have been conducted that involve the use of UK citizens. Additionally, there have been no experimental studies that have investigated welfare message framing and its influence on the public's perceptions of cow–calf separation. Although Hotzel et al. (4)'s study involved welfare information, it is difficult to ascertain which welfare factor, if any, was causing a behaviour change. Naturalness within farming and achieving good welfare for farm animals are two key public concerns relating to livestock production (18). By asking participants to consider welfare narratives within the context of cow–calf separation, those who perceive the practice as unnatural may face indirect trade-offs between these concerns, revealing insights into which issue is of greater importance to the public. Finally, by changing welfare message frames to investigate decision-making changes, it will provide further information on which components of animal welfare are of greater consideration to the public; a concept which has previously been unexplored.

Factorial survey experiments are increasingly employed as a method for assessing decision-making behaviours. To date, they have been used for measuring decision-making in security (22), criminology (23) and medicine (24). In an agricultural context, they have been used to investigate veterinarians' antibiotic prescribing behaviours (25) and producers' risk perception of bovine tuberculosis (26). However, factorial surveys have not yet been applied to citizens' decision-making behaviours in an agricultural context. In a factorial survey, participants are presented with textual scenarios, vignettes, that systematically vary in the factors that are presented within them (27). By manipulating the factors that appear in each vignette, it is possible to determine the influence of each factor on the respondent's decisions. Assessing the vignettes as a whole, which are often complex and comprised of multiple pieces of information, is more aligned with real-life decision making due to the trade-offs that occur when assessing the vignette text. As a result, the respondent's decisions are captured with greater validity and reliability (28). The aim of this study was to assess the influence of welfare message framing on the public's decisions on cow–calf separation, namely their acceptance of the practice and their assessment of the welfare impact, through the presentation of welfare narratives pertaining to the calf.

## 2 Materials and methods

### 2.1 Survey design

To investigate citizens' decision-making behaviours in the context of cow–calf separation, a 2 × 2 × 2 × 2 × 2 factorial survey design was used (29). In the survey, respondents were presented with a series of eight vignettes which mimicked the potential aftermath of a calf's life following its separation from the cow. Each vignette consisted of five factors, with each factor having two possible levels. Although the order in which the factors appeared within the vignette remained the same, the level that appeared was experimentally varied. A total of 32 unique vignette scenarios were possible. From this sample, a resolution V D-efficient design (30) was used to randomly select 16 unique vignette combinations, which were then split randomly into two blocks of eight vignettes. This approach was used as it is recommended that no more than 10 vignettes should be rated per respondent (31). Half of the survey respondents received the first block of vignettes and the other half of the respondents received the second block of vignettes.

#### 2.1.1 Respondent-level predictor variables

At the beginning of the survey, respondents were asked a series of closed questions to gain insight into the sociodemographic factors of the respondent population. The resulting characteristics constituted the respondent-level predictor (i.e., independent) variables in the study. Respondents were asked questions about their gender, their age, the region of the UK in which they live, the highest level of education they have achieved, their dietary preference, the type of areas they have lived in for most of their life, and their take-home household income. Respondents were also asked about their links to the dairy industry, the extent of their knowledge on dairy farming and how it operates, and their main source of information on the dairy sector. Finally, respondents were asked whether they were aware that calves were separated from the cow at birth on farms in the UK and the extent to which they agreed with this practice prior to completing the vignette evaluations.

#### 2.1.2 The vignette scenario, vignette-level predictor variables, and dependent variables

Before completing the vignette section of the survey, respondents were presented with a scenario describing a farmer who had recently implemented cow–calf separation within 24 h of birth. In this scenario, calves were separated from their biological dam, were not reared with a foster cow, and were monitored for several days following separation to assess the effects of this management practice. This information was intended to contextualise the vignettes, which subsequently offered detailed insight into the calf's life and the effects of early cow–calf separation on its welfare. The vignette-level predictor variables were chosen to represent the Five Domains Model, a widely accepted framework for assessing animal welfare (32). These domains are: (1) Nutrition; (2) Physical Environment; (3) Health; (4) Behavioural Interactions; and (5) Mental State (32). The levels for each variable represent both a positive and a negative welfare outcome that could occur following separation within each of the five domains. These outcomes were chosen based on evidence from the literature to ensure that the welfare narratives were grounded in empirical research, and are presented in Table 1. The phrasing of the outcomes was guided by feedback obtained from pilot testing, which was carried out with five individuals who varied in their knowledge of the dairy industry, to ensure that the variables were clear and easy to understand. Where feedback indicated that a variable was not readily understood, a concise description of its relevance to welfare was incorporated into the phrasing to improve clarity. The revised phrasing was reviewed by the feedback provider to confirm that the change adequately addressed the concern.

**Table 1:** The variables included within the factorial survey vignettes.

Predictor variable	Level		Literature reference
Nutrition	1	The calf has a low daily milk intake due to smaller volumes being available	(62)
	2	The calf has consumed an adequate amount of good quality colostrum, administered by the farmer, which is important for establishing early-life immunity	(63)
Physical environment	1	The calf has been avoiding novel objects in the pen	(64)
	2	The calf has greater space availability due to the absence of the cow	(65)
Health	1	The calf has been recorded as having a below average daily weight gain	(14)
	2	The calf has not been diagnosed with Failure of Passive Transfer, a condition causing lifelong susceptibility to diseases	(66)
Behavioural interactions	1	The calf has been demonstrating abnormal behaviours such as tongue rolling and sucking on pen fixtures	(67, 68)
	2	The calf has been socially interacting with other calves in the pen, forming friendship bonds	(65, 69)
Mental state	1	The calf has exhibited signs of insecurity due to the lack of maternal bonding	(15)
	2	The calf has exhibited no signs of separation stress	(12)

1 = A negative outcome within the welfare domain; 2 = A positive outcome within the welfare domain.

After each vignette, respondents were asked to answer two questions based only on the information that was provided in the vignette (Figure 1). These were:

1. The extent to which they agree with the farmer's choice to separate the calf from the cow given the outcome (Outcome A), where −5 = definitely do not support and +5 = definitely do support;
2. The extent to which they believe the calf's welfare has been affected by the separation from the cow given the outcome (Outcome B), where −5 = welfare has been negatively affected and +5 = welfare has been positively affected.

**Figure 1:**
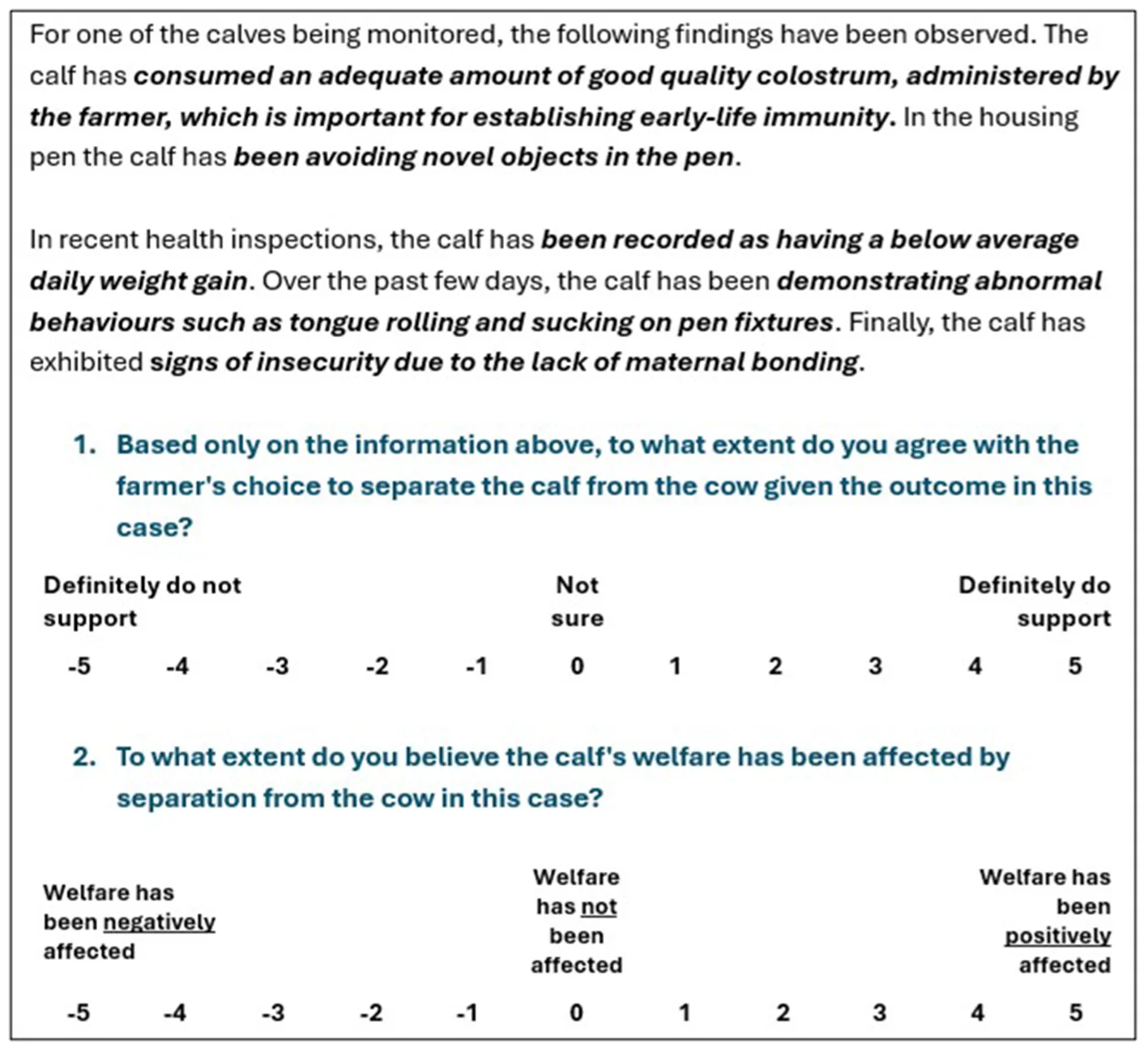
An example vignette used in the factorial survey, illustrating the vignette-level predictor variables (shown in bold) and the two dependent outcome variables.

The respondents could review and edit their responses throughout the survey; however, they were instructed to base their answers to the two outcome questions solely on the information presented in the preceding vignette. An 11-point scale was chosen in accordance with previous factorial survey research, as smaller scales can result in response censoring whilst larger scales can lead to overwhelm and poor scale use (31). Additionally, the use of an 11-point scale provides respondents with a clear midpoint through which uncertainty can be expressed (33), thereby strengthening the validity of the estimated effects of the vignette variables on the two outcomes.

### 2.2 Participant recruitment

Potentia Insight, a market research company based in the United Kingdom, was used to recruit participants in the study. The company distributed the online survey on behalf of the University of Nottingham School of Veterinary Medicine and Science in March 2025 and aimed to recruit at least 500 sociodemographically diverse citizens aged 18 years and older from across the UK. In the context of this study, a citizen was defined as a native or naturalised member of the United Kingdom. The term “citizen” was used in preference to “consumer” to reflect the inclusion of participants adhering to vegan or dairy-free diets, as these individuals would not be considered consumers of the dairy industry. Respondents who completed the survey were offered an incentive as per the company's standard incentive scheme. The acquired sample was nationally representative with respect to gender, age and geographical region. It was not possible to obtain a nationally representative sample in relation to the remaining respondent-level variables involved in the study as official national statistics for these variables are not available. The survey took approximately 10 min to complete. An example survey can be found in Supplementary material 1.

The required sample size to ensure adequate statistical power during the analysis was calculated using G^*^Power: (70) for a linear multiple regression analysis with up to 17 predictor variables (34). A small effect size of 0.02 and a power of 0.95 was chosen in line with previous literature (25). To achieve adequate power, a sample of 1,467 responses was required. However, as the vignette is the unit of analysis, and each participant rates eight vignettes, a sample size of 183 respondents was required for the analysis.

### 2.3 Ethical review

Ethical approval for the study was granted by the University of Nottingham School of Veterinary Medicine and Science Ethics Committee (no. 3852 230516). The respondents were informed that the survey was anonymous and that their participation was voluntary, with any data generated being used solely for research purposes. Consent to participate in the survey was indicated by the respondent continuing to proceed with the survey after being shown the consent form prior to the start of the survey questions. Compliance with General Data Protection Regulation 2016/679 was outlined to participants through a privacy notice that was embedded within the consent form.

### 2.4 Statistical analysis

#### 2.4.1 Data processing and preliminary analysis

Once collected, the data was checked for errors and processed using Stata 15.1 (StataCorp, College Station, TX, USA). Preliminary analysis of the data involved summarising the descriptive characteristics of the respondent population as well as performing univariable analysis to investigate associations between the predictor variables and the two survey outcomes. Where appropriate, response options for some of the predictor variables were recoded as a combined response option to assist with data handling. For both survey outcomes, the original −5 to +5 rating scale was recoded to a 1–11 scale. Both survey outcomes were analysed as continuous variables as suggested for factorial survey research (31).

#### 2.4.2 Multilevel modelling

Mixed-effects multilevel linear models were built for both outcomes using Stata 15.1 (StataCorp, College Station, TX). For both models, a bottom-up building strategy was used as described by Douglas (35). Initially, univariable analysis was undertaken to identify candidate predictor variables associated with each outcome. Variables that demonstrated evidence of an association during the univariable analysis (*p* ≤ 0.05) were selected for inclusion in the multilevel mixed effects models. This approach was used given the exploratory nature of the analysis and to reduce model complexity due to the large number of predictors included in the study. Interaction effects were not explored in the multilevel models as there were no strong prespecified theoretical or biological hypotheses that indicated the presence of meaningful two-way interactions. As mentioned by Doidge et al. (25), the developed random intercept models containing both the vignette-level and respondent-level predictors can be expressed as:


$$\begin{array}{l} y_{ab}=\;\beta_{0ab}+\;\beta_{f1}\;vignette\;predictors_{ab}\\ \;+\;\beta_{g1}respondent\;predictors_{b}+\;u_{0b}+\;e_{0ab} \end{array}$$

where *y*_*ab*_ is the level of agreement with the choice the separate the calf (Outcome A) or the extent to which the calf's welfare has been affected by the separation (Outcome B) for vignette *a*, rated by respondent *b*; β_0ab_ is the intercept; β_f1_ is the beta-coefficient for the effect a unit increase in the predictor *f*_ab_ has on the outcome; β_g1_ is the beta-coefficient for the effect a unit increase in the predictor *g*_b_ has on the outcome; and *u*_0b_ and *e*_0ab_ are the respondent-level and vignette-level random effects, respectively.

#### 2.4.3 Model checks

A series of model checks were performed to evaluate the final fit of the models. Inspection of the residuals at every level was performed to check their adherence to a normal distribution and to identify the presence of homoscedasticity. To compare the performance of the full model compared to the null model for both outcomes, Akaike's Information Criterion (AIC) was calculated. To determine the level of variance in the vignette ratings that is the result of differences between the respondents, as opposed to differences within respondents across vignettes, the variance partitioning coefficient (VPC) was calculated. Finally, the mean absolute error (MAE) was calculated to identify the model's predictive accuracy. All model checks were carried out in Stata 15.1.

## 3 Results

### 3.1 Respondent demographics

A total of 503 respondents completed the survey, resulting in 4,024 vignette evaluations. A summary of the respondent characteristics can be found in Supplementary material 2. Of the respondents, 50.1% were female. Over half (55.7%) of the respondents were aged 45 and over, with just under half (40.2%) possessing qualifications at a university level. The majority of the respondents had no links with the dairy industry (57.1%) and possessed no (30.6%) to little (61.4%) knowledge on dairy farming. Over half (53.5%) of the respondents were not aware that cow–calf separation occurred on dairy farms in the United Kingdom prior to completing the vignette evaluations. Furthermore, 35.6% of participants stated that they neither supported nor opposed this practice prior to answering the vignette scenarios.

### 3.2 Outcome A: agreement with the choice to separate the calf

Model 1, a random intercept model demonstrating the respondents' agreement with cow–calf separation (Outcome A), is presented in Table 2. When compared to a negative welfare outcome, a positive outcome within the physical environment, health, behavioural interactions, and mental state welfare domains increased the respondents' agreement with cow–calf separation. For the variables “the calf has been socially interacting with other calves in the pen, forming friendship bonds” (β = 0.701; CI = 0.561, 0.842) and “the calf has exhibited no signs of separation stress” (β = 0.663; CI = 0.504, 0.823), the respondents' agreement with the practice increased by over half a point, whilst the variable “the calf has not been diagnosed with Failure of Passive Transfer” (β = 0.477; CI = 0.351, 0.604) increased the respondents' agreement with the separation by under half a point. The variable “the calf has greater space availability in the pen” had a smaller, but significant, influence on the respondents' agreement (β = 0.106; CI = 0.003, 0.210). The variable “the calf has consumed an adequate amount of good quality colostrum,” a positive welfare outcome within the nutrition welfare domain, did not significantly influence the respondents' agreement with cow–calf separation when compared to a negative welfare outcome (β = 0.067; CI = −0.050, 0.185). Respondents who “strongly supported” (β = 1.348; CI = 0.727, 1.969) or “somewhat supported” (β = 1.046; CI = 0.519, 1.572) cow–calf separation prior to evaluating the vignettes had a higher agreement rating than participants who did not have an opinion on the practice. Finally, the respondents' prior awareness of cow–calf separation did not influence their agreement with the practice (β = −0.239; CI = −0.661, 0.182).

**Table 2:** A random intercept model to explain the level of agreement demonstrated by the respondents with the choice to separate the calf from the cow in the vignette evaluations (Outcome A).

Outcome A	Model 1	
	β	SE
Nutrition welfare domain		
The calf has a low daily milk intake due to smaller volumes being available	Ref	Ref
The calf has consumed an adequate amount of good quality colostrum, administered by the farmer, which is important for establishing early-life immunity	0.067	0.060
Physical environment welfare domain		
The calf has been avoiding novel objects in the pen	Ref	Ref
The calf has greater space availability due to the absence of the cow	0.106^*^	0.053
Health welfare domain		
The calf has been recorded as having a below average daily weight gain	Ref	Ref
The calf has not been diagnosed with Failure of Passive Transfer, a condition causing lifelong susceptibility to diseases	0.477^***^	0.064
Behavioural interactions welfare domain		
The calf has been demonstrating abnormal behaviours such as tongue rolling and sucking on pen fixtures	Ref	Ref
The calf has been socially interacting with other calves in the pen, forming friendship bonds	0.701^***^	0.072
Mental state welfare domain		
The calf has exhibited signs of insecurity due to the lack of maternal bonding	Ref	Ref
The calf has exhibited no signs of separation stress	0.663^***^	0.081
Age group		
44 and under	Ref	Ref
45 and over	−0.422^*^	0.209
Knowledge of the dairy industry		
I do not know anything about dairy farming	Ref	Ref
I know a little bit about dairy farming because of social media and/or mainstream news	−0.517^*^	0.234
I know a little bit about dairy farming because I read agricultural articles, attend open farms, or attend livestock/dairy events	−0.102	0.291
I know a lot about dairy farming by possessing a qualification relevant to agriculture, working with dairy cattle myself, or having family links to farming	−0.078	0.369
Type of area lived		
Urban	Ref	Ref
Suburban	−0.479^*^	0.220
Rural	−0.473	0.269
Mixture	−1.204^***^	0.315
Prior agreement with cow–calf separation		
Neither support nor oppose	Ref	Ref
Strongly support	1.348^***^	0.317
Somewhat support	1.046^***^	0.269
Somewhat oppose	−1.065^***^	0.252
Strongly oppose	−2.428^***^	0.290

Intercept	5.846	0.112
Log pseudolikelihood	−8,628.139	
AIC	17,342.280	
VPC	0.625	
MAE	1.979	

^*^P ≤ 0.05; ^***^P ≤ 0.001.

For the variables “being 45 and over” (β = −0.422; CI = −0.832, −0.011), “I know a little bit about dairy farming because of social media and/or mainstream news” (β = −0.517; CI = −0.976, −0.058), “I have lived in suburban areas for most of my life” (β = −0.479; CI = −0.910, −0.049), and “I have lived in a mixture of areas for most of my life” (β = −1.204; CI = −1.821, −0.587), the respondents' agreement with cow–calf separation decreased. Additionally, respondents who “strongly opposed” (β = −2.428; CI = −2.997, −1.860) or “somewhat opposed” (β = −1.065; CI = −1.558, −0.572) cow–calf separation prior to evaluating the vignettes had a lower agreement rating than participants who did not have an opinion on the practice.

Overall, the physical environment, health, behavioural interactions and mental state welfare domains were influential on the respondents' judgements, with a positive outcome within these domains increasing the participants' agreement with cow–calf separation. Additionally, respondents who expressed support for the practice prior to completing the survey were also more likely to agree with the practice throughout the survey. However, respondents who were older, who had little knowledge of the dairy industry through social media or mainstream news, and who lived in suburban or a mixture of areas were more likely to disagree with cow–calf separation. Furthermore, respondents who expressed opposition to the practice prior to completing the survey were more likely to disagree with the practice throughout the survey. These findings have been outlined in Figure 2.

**Figure 2:**
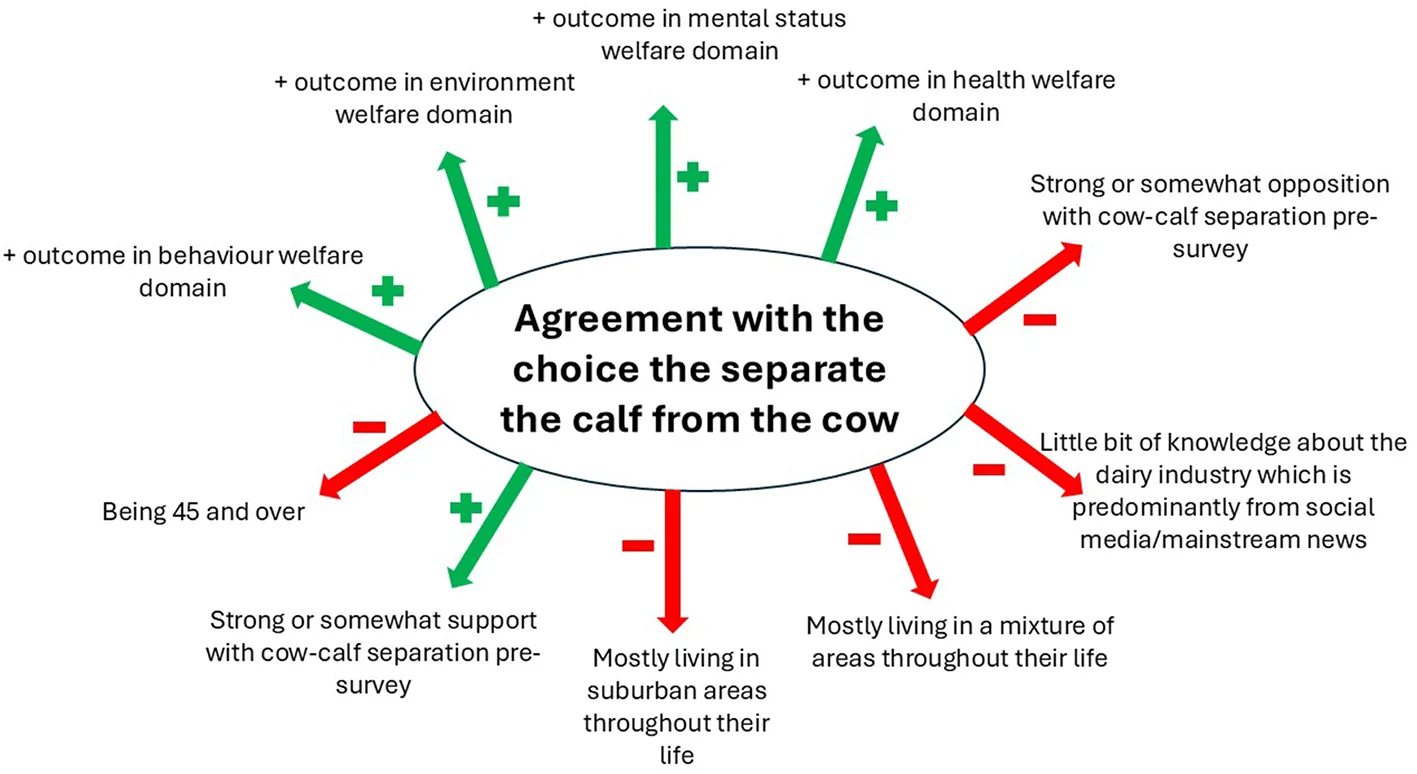
Significant factors associated with the agreement with cow–calf separation (+ and –signs indicate the direction of associations).

### 3.3 Outcome B: impact of the separation on the calf's welfare

Model 2, a random intercept model illustrating the respondents' perception of the welfare impact of cow–calf separation on the calf (Outcome B), is presented in Table 3. When compared to a negative welfare outcome, a positive outcome within the health, behavioural interactions, and mental state welfare domains resulted in the welfare impact being perceived more positively by respondents. For the variables “the calf has been socially interacting with other calves in the pen, forming friendship bonds” (β = 0.762; CI = 0.609, 0.914) and “the calf has exhibited no signs of separation stress” (β = 0.805; CI = 0.629, 0.981), the respondents' positive perception of the welfare impact increased by over half a point, whilst the variable “the calf has not been diagnosed with Failure of Passive Transfer” (β = 0.479; CI = 0.334, 0.624) increased their positive perception of the welfare impact by under half a point. The variables “the calf has consumed an adequate amount of good quality colostrum” (β = −0.042; CI = −0.174, 0.090), a positive welfare outcome within the nutrition welfare domain, and “the calf has greater space availability in the pen” (β = −0.007; CI = −0.113, 0.098), a positive welfare outcome within the physical environment welfare domain, did not significantly influence their perception of the welfare impact when compared to a negative welfare outcome. Respondents who “strongly supported” (β = 1.578; CI = 0.965, 2.191) or “somewhat supported” (β = 0.997; CI = 0.473, 1.521) cow–calf separation prior to evaluating the vignettes were more likely to perceive the welfare impact more positively than those who had no opinion on the practice. However, the respondents' prior awareness of cow–calf separation did not significantly influence their perception (β = −0.223; CI = −0.644, 0.199). Finally, “being pescetarian” (β = 0.812; CI = 0.176, 1.448) also had a positive influence on their perceptions.

**Table 3:** A random intercept model to explain the respondents' positive or negative welfare impact rating following separation of the calf from the cow in the vignette evaluations (Outcome B).

Outcome B	Model 2	
	β	SE
Nutrition welfare domain		
The calf has a low daily milk intake due to smaller volumes being available	Ref	Ref
The calf has consumed an adequate amount of good quality colostrum, administered by the farmer, which is important for establishing early-life immunity	−0.042	0.067
Physical environment welfare domain		
The calf has been avoiding novel objects in the pen	Ref	Ref
The calf has greater space availability due to the absence of the cow	−0.007	0.054
Health welfare domain		
The calf has been recorded as having a below average daily weight gain	Ref	Ref
The calf has not been diagnosed with Failure of Passive Transfer, a condition causing lifelong susceptibility to diseases	0.479^***^	0.074
Behavioural interactions welfare domain		
The calf has been demonstrating abnormal behaviours such as tongue rolling and sucking on pen fixtures	Ref	Ref
The calf has been socially interacting with other calves in the pen, forming friendship bonds	0.762^***^	0.078
Mental state welfare domain		
The calf has exhibited signs of insecurity due to the lack of maternal bonding	Ref	Ref
The calf has exhibited no signs of separation stress	0.805^***^	0.090
Age group		
44 and under	Ref	Ref
45 and over	−0.413^*^	0.205
Knowledge of the dairy industry		
I do not know anything about dairy farming	Ref	Ref
I know a little bit about dairy farming because of social media and/or mainstream news	−0.648^**^	0.222
I know a little bit about dairy farming because I read agricultural articles, attend open farms, or attend livestock/dairy events	−0.632^*^	0.283
I know a lot about dairy farming by possessing a qualification relevant to agriculture, working with dairy cattle myself, or having family links to farming	−0.288	0.389
Links to the dairy industry		
Has some links to the dairy industry	Ref	Ref
Has no links to the dairy industry	−0.507^*^	0.227
Dietary preference		
Omnivore	Ref	Ref
Dairy-free	0.343	0.319
Pescetarian	0.812^*^	0.325
Flexitarian	−0.054	0.271
Non-meat eating (vegetarians and vegans)	0.583	0.302
Type of area lived		
Urban	Ref	Ref
Suburban	−0.392	0.206
Rural	−0.527^*^	0.260
Mixture	−1.229^***^	0.297
Prior agreement with cow–calf separation		
Neither support nor oppose	Ref	Ref
Strongly support	1.578^***^	0.313
Somewhat support	0.997^***^	0.267
Somewhat oppose	−0.811^***^	0.232
Strongly oppose	−1.977^***^	0.271

Intercept	5.539	0.108
Log pseudolikelihood	−8864.623	
AIC	1,7815.250	
VPC	0.566	
MAE	2.019	

^*^P ≤ 0.05; ^**^P ≤ 0.01; ^***^P ≤ 0.001.

For the variables “being 45 and over” (β = −0.413; CI = −0.814, −0.011 ), “I know a little bit about dairy farming because of social media and/or mainstream news” (β = −0.648; CI = −1.083, −0.214), “I know a little bit about dairy farming because I read agricultural articles or attend livestock events” (β = −0.632; CI = −1.187, −0.077), “I have lived in rural areas for most of my life” (β = −0.527; CI = −1.036, −0.018), and “I have lived in a mixture of areas for most of my life” (β = −1.229; CI = −1.811, −0.647), the respondents' perception of the welfare impact was more negative. Additionally, respondents who “strongly opposed” (β = −1.977; CI = −2.509, −1.445) or “somewhat opposed” (β = −0.811; CI = −1.267, −0.356) cow–calf separation prior to evaluating the vignettes were more likely to perceive the impact more negatively than those who had no opinion on the practice. Finally, the variable “I have no links with the dairy industry” (β = −0.507; CI = −0.953, −0.062) was found to have a significant negative impact on the respondents' perceptions.

Overall, the health, behavioural interactions, and mental state welfare domains were influential on the respondents' ratings, with a positive outcome within these domains having a positive influence on how respondents perceived the welfare impact. Additionally, respondents who were pescetarian and those that expressed support for cow–calf separation prior to completing the survey were also more likely to have a positive perception of the welfare impact. However, respondents who were older, who had little knowledge of the dairy industry through social media, mainstream news, or agricultural sources, who had no links to the dairy industry, and who lived in rural or a mixture of areas were more likely to have a negative perception of the welfare impact. Furthermore, respondents who expressed opposition to cow–calf separation prior to completing the survey were more likely to have a negative perception of the welfare impact. These findings have been outlined in Figure 3.

**Figure 3:**
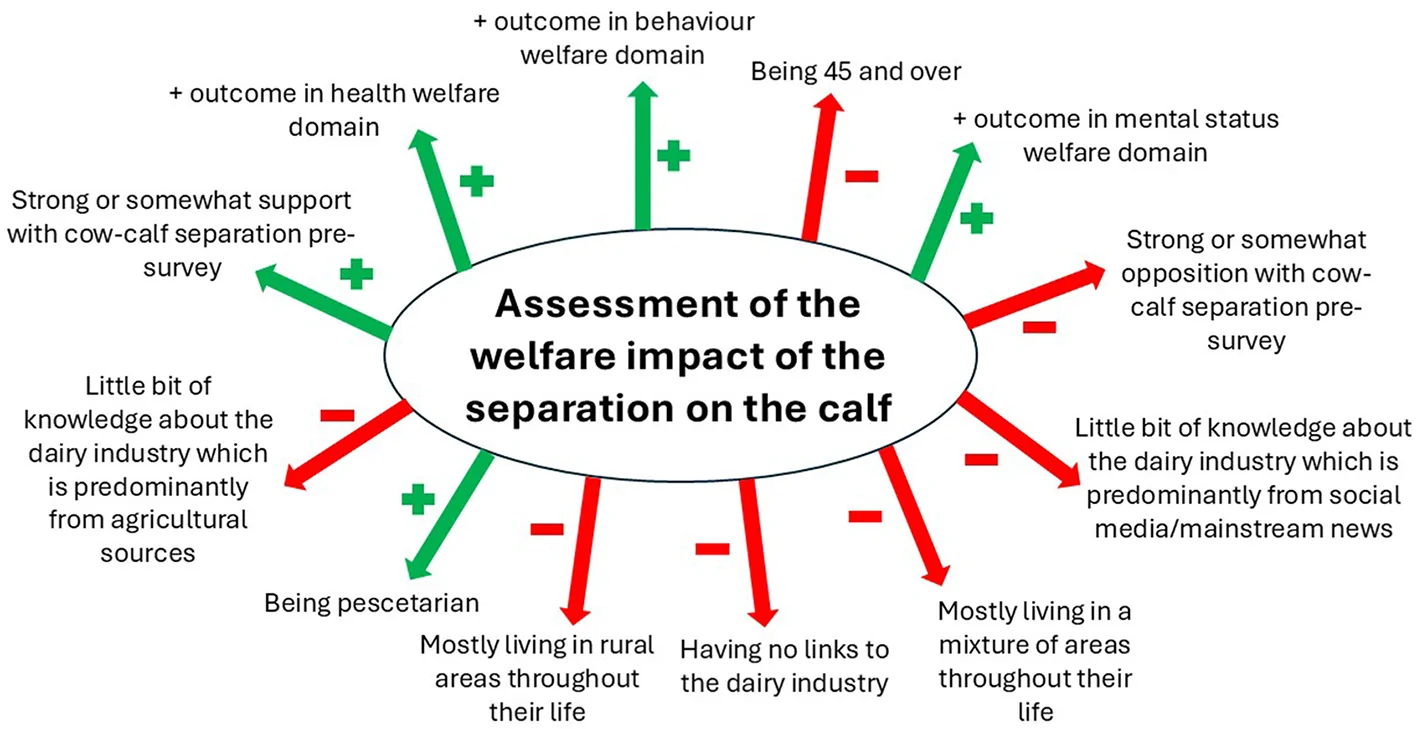
Significant factors associated with the perception of the welfare impact that the separation had on the calf (+ and –signs indicate the direction of associations).

### 3.4 Model fit

For both outcomes, inspection of the residuals at all levels indicated a normal distribution. However, there was evidence of heteroscedasticity in both models; therefore, the models were rerun using robust standard errors to control for this. The VPC for the models highlighted that 56.6%−62.5% of the decisions made in the survey were attributed to differences between the respondents who completed the survey, rather than differences between the vignettes themselves. The MAE demonstrated a decrease between the null model, containing the random intercept only, and the full model, containing the random intercept as well as the vignette-level and respondent-level predictors, for both outcomes, indicating improved predictive accuracy of the models. Finally, the AIC was lower for the full models for both outcomes compared to the null models, demonstrating improved model fit.

## 4 Discussion

To the authors' knowledge, this is the first study to use a factorial survey approach to understand citizens' decision-making in relation to cow–calf separation, and how welfare message framing influences their perceptions of this practice. Our results identified that the behavioural and mental state welfare domains were the most influential in increasing the respondents' agreement with the practice as well as resulting in the welfare impact being viewed more positively, compared with the other welfare domains. This suggests that the social and mental wellbeing of calves has a comparatively stronger role in citizens' welfare evaluations than clinical indicators of wellbeing such as health and nutrition.

Among the five welfare domains, a positive outcome within the mental state welfare domain exhibited the largest effect on the respondents' perception of the welfare impact, and the second largest effect on the participants' agreement with cow–calf separation. The prominence of this domain suggests that the public evaluates this practice through the lens of the calf's subjective experiences. Within the Five Domains framework, the mental state domain represents the cumulative affective consequences arising from conditions within the remaining domains (32); therefore, the larger effect size of the mental state domain highlights that respondents emphasised experiential aspects of welfare over appropriate physiological functioning in relation to cow–calf separation. Previous studies have similarly identified that citizens' concerns for animal emotions and separation stress were central reasons for opposing cow–calf separation (3, 4). The strong influence of the mental state domain in the present study is therefore unsurprising, particularly as the variable representing this welfare domain stated that “the calf has exhibited no signs of separation stress.” This concern for the emotional wellbeing of calves is aligned with the realities of cow–calf separation, where biological stress, through increased cortisol levels (36), and behavioural stress, through vocalisations (37), have been documented following separation. Additionally, separation stress has been associated with pessimistic judgement biases in calves, which are interpreted as indicators of negative affective states (15). Together, this highlights that citizens' concerns for a calf's emotional experiences during separation are not simply intuitive, but are reflective of welfare outcomes that have been investigated in the literature.

A positive outcome within the behavioural interactions welfare domain exhibited the greatest influence on the respondents' agreement with cow–calf separation, and the second largest influence on their perception of the welfare impact, compared to the other welfare domains. The strength of the influence of this domain suggests that citizens place considerable value on providing calves with the opportunity to engage in social interactions and engage in behaviours that are intrinsically rewarding. This finding is consistent with previous research which demonstrated that citizens associate animal welfare with the ability to express natural behaviours (18). It also highlights an area of convergence between citizens' welfare priorities and contemporary welfare frameworks, which recognise the importance of behavioural interactions as a source of positive welfare (32). As a result, the prominence of this welfare domain provides further evidence that the public conceptualises animal welfare beyond good physiological functioning in relation to cow–calf separation. From an ethological perspective, calves are highly social animals that are motivated to interact with conspecifics and engage with their surrounding environment (38). The provision of social interactions facilitates the formation of social bonds (39), supports the development of social competence (40), and promotes the expression of play behaviours (41). Resultingly, the provision of social interactions may have been perceived as an indicator of good welfare due to it enabling calves to engage in self-motivated behaviours. However, an important limitation of the variable used to represent this welfare domain is acknowledged. Unlike the other variables, this variable used emotive language through the word “friendship.” It is therefore possible that the respondents' judgements were influenced by the affect heuristic, a type of cognitive bias whereby decisions are shaped by emotional responses rather than deliberate reasoning (42). The term “friendship” may have evoked feelings of attachment, companionship and affection which could have increased the perceived importance of this welfare outcome.

A positive welfare outcome within the health domain was influential in respondents' ratings of both outcomes; however, its effect was smaller than those associated with the behavioural interactions and mental state domains. Previous research has identified that citizens' concerns for calf health were another consideration when evaluating cow–calf separation (1, 20), aligning with the findings of this study. However, the comparatively smaller effect size of this welfare domain in the present study suggests that the respondents did not solely conceptualise welfare, in the context of cow–calf separation, as good biological functioning. This aligns with contemporary welfare frameworks that increasingly acknowledge social and mental wellbeing as critical components of animal welfare, in addition to physiological wellbeing (32). It may also highlight that respondents acknowledge that calves can remain clinically healthy whilst simultaneously experiencing negative emotional states or restrictions on behavioural expression (43), which could provide further reasoning for the smaller effect size of the health domain compared with the behavioural interactions and mental state domains. From an ethological perspective, the significance of good health extends beyond the prevention of suffering, as healthy calves are better able to engage with their environment and participate in social interactions (44, 45). An alternative explanation for the reduced effect of this welfare domain relates to the use of agricultural jargon within the wording of the vignette. The term “Failure of Passive Transfer” may not have been connected to outcomes such as reduced growth (46) and increased susceptibility to morbidity and mortality (47). However, the vignette clarified that this condition resulted in lifelong disease susceptibility, and feedback from pilot testing did not indicate substantial difficulties in understanding the description; therefore, the effects of this terminology are likely to have been limited. Nevertheless, it highlights the importance of considering technical language when designing public perception studies.

A positive outcome within the physical environment welfare domain had the smallest significant influence on the respondents' agreement with cow–calf separation, compared to the other significant welfare domains, and exhibited no influence on their perception of the welfare impact. This finding is surprising, particularly as citizens have expressed a desire for farmed animals to be housed with adequate space due to their growing concern for intensification within farming (48). However, this finding suggests that respondents perceived cow–calf separation as a social welfare issue, with the consequences for calves' behavioural opportunities and affective experiences exerting greater influence on their judgements than environmental conditions. It is also possible that the environmental consequences associated with cow–calf separation were less immediately visible to the respondents compared to the other welfare domains. As no additional information was provided on the benefits of increased space allowance, respondents may not have recognised its potential contribution to welfare through improved comfort and increased locomotor behaviours (49, 50). The variables chosen to represent the environment domain introduce a potential framing bias, which is an acknowledged limitation. Respondents were asked to choose between avoiding novel objects and increased space allowance due to the absence of the dam when considering this welfare domain. As the welfare outcomes associated with increased space provision are not comparable to the benefits of maternal contact, it is possible that respondents were considering two suboptimal welfare scenarios.

Several respondent characteristics also influenced the respondents' evaluations of the vignettes. Participants aged 45 years and over were less likely to agree with cow–calf separation and perceived the welfare impact more negatively than younger participants which is in line with previous studies (1, 4). However, previous research has also shown that older generations are more accepting of contemporary farming practices than younger generations (51). Interestingly, respondents adhering to a pescetarian diet perceived the welfare impact of the practice more positively, a finding that has not been identified in the literature before. Previous studies, instead, have demonstrated that vegetarians favoured later separation (4); a finding that was not replicated in this study. In contrast to previous studies (1, 4), gender did not influence the respondents' evaluations of the vignettes, despite research suggesting that females are more sensitive to animal welfare topics (52).

Respondents who lived in suburban areas and those who have lived in a mixture of areas were less likely to agree with cow–calf separation, and respondents who lived in rural areas and those who have lived in a mixture of areas perceived the welfare impact more negatively. It is possible that these individuals are more exposed to dairy farming practices, and their impacts, on a day-to-day basis than individuals living in urban regions, with the latter becoming increasingly disconnected from agricultural practices (53). Participants who did not have links to the dairy industry perceived the welfare impact of the separation more negatively, which has been identified in other studies (1, 4). It has been identified that such individuals have a less positive view on farmers (54) and perceive the quality of life of farm animals more negatively (51) which could explain this finding. Finally, individuals who possessed little knowledge of the dairy industry were less likely to agree with the practice and perceived the welfare impact more negatively, which is aligned with previous findings (1). Interestingly, individuals who possessed dairy industry knowledge predominantly from social media or mainstream news demonstrated more negative ratings in their vignette evaluations than those who possessed no knowledge of the dairy industry. This highlights the increasing influence of social media on citizens' beliefs, particularly on topics in which they are unfamiliar (55), which is often imbalanced and a large contributor to misinformation when reporting on agricultural topics (56, 57).

Respondents who strongly agreed or disagreed with cow–calf separation prior to completing the survey were more likely to rate the two study outcomes more positively or negatively, respectively. This aligns with previous research, where increasing information provision (4) and visits to dairy farms (21) failed to improve respondents' agreement with the practice and in some cases worsened their perceptions. This suggests that although respondents placed different values on the welfare domains when evaluating the vignettes, their prior opinion also shaped their judgements on this practice. It is possible that this is caused by confirmation bias, where greater emphasis is placed on vignette factors that align with their initial position on the practice (58). The respondents' moral intuition may have also influenced their judgements during the survey, where the choices made are guided by an individual's feelings of appropriateness about a situation rather than by a conscious decision process (59). However, as naturalness within livestock production remains a key concern amongst citizens (18), the perceived unnaturalness of cow–calf separation could have been a driver for the respondents' disagreement with the practice when evaluating the vignettes. Although the vignettes depicted welfare outcomes across the Five Domains, it is possible that any positive welfare outcomes did not fully offset the respondents' concerns around the unnaturalness of the practice. Therefore, by asking participants to evaluate welfare narratives within the context of a perceived unnatural management practice, it may have resulted in an indirect trade-off between the welfare outcomes for the calf and the perceived naturalness of the practice.

Unlike previous studies, where the majority of respondents disagreed with cow–calf separation (1, 3), the relative majority of respondents in the present study indicated a neutral stance on cow–calf separation prior to evaluating the vignettes. As over half of the respondents were unaware of cow–calf separation, and its occurrence on dairy farms in the UK, prior to completing the survey, the respondents' neutrality to the practice is therefore unsurprising. Professed ignorance of the public on agricultural practices has been identified previously (60) and is continually exacerbated by urbanisation and the subsequent disconnect of citizens from farming (53). Consequently, respondents may have relied on anthropomorphic reasoning (60) and decision-making heuristics (42) to combat their lack of awareness and lack of opinion on the practice when making their judgements.

Several limitations to this study are acknowledged. The use of UK citizens may limit the study's applicability to citizens in other countries, particularly where cultural differences exist or where attitudes towards the agricultural sector differ. Heteroscedasticity was identified in the model during analysis; however, this was controlled for using robust standard errors (61) to ensure any conclusions made from the data were accurate. Variable selection for inclusion in the multilevel models was informed by univariable significance; therefore, it is possible that some important confounders were not identified if they did not demonstrate independent univariable significance. However, all vignette-level variables, which represented the welfare domains, were retained in both models using this approach, which were the primary focus of the research question. Respondent-level variables, such as “being pescetarian” and “I have lived in a mixture of areas for most of my life” influenced respondents' ratings; however, only a small percentage of the respondents belonged to each category. Consequently, care must be taken when drawing conclusions from these results as the sample is likely non-representative.

The framing of the scenario that precedes the vignettes described a farmer who had recently implemented early cow–calf separation as a new management practice, rather than presenting the practice as routine within the industry. This may have led respondents to view the farmer as deliberately choosing early separation over prolonged cow–calf contact, which could have influenced their judgements if they considered the former as a lower-welfare option. However, this interpretation is unlikely to have been universal amongst the respondents. Prior to completing the study, some participants expressed support for cow–calf separation, suggesting that the practice was not viewed negatively by all the respondents. Resultingly, it cannot be assumed that all the participants viewed early separation as inferior to prolonged contact from a welfare perspective.

The findings of the study are specific to the outcomes used to represent the welfare domains; therefore, the relative importance of the welfare domains could change where different outcomes are present. The language used in the vignette scenarios included both clinical terminology and anthropomorphic terms which could have influenced the effect of the predictors on the respondents' vignette ratings. Similarly, it is possible that the relevance of certain vignette predictors on calf welfare was not well understood, further influencing their effect on the participants' judgements. However, the terminology used and the extent to which variables were explained were guided by feedback from pilot testing to ensure that the vignettes were both clear and concise.

The vignettes were long and consisted of complex information which could have exposed the respondents to survey fatigue and could have resulted in a lack of care when completing the survey. However, all data was cleaned during the analysis to control for errors in the dataset. Finally, as this study used a quantitative methodology, we do not fully understand the reasoning behind participants' evaluations in the survey. Therefore, qualitative exploration is required to understand the data beyond what is discussed here.

## 5 Conclusion

This study used a factorial survey approach to understand how welfare message framing influences UK citizens' perceptions of cow–calf separation. Four of the animal welfare domains significantly influenced the respondents' agreement with cow–calf separation as well as their evaluation of the welfare impact of this practice on the calf. These were environment, health, behavioural interactions and mental state. Several sociodemographic factors also significantly influenced the respondents' ratings. However, the preconceived beliefs of the respondents on this practice were also influential on their decision-making process, suggesting that the unnaturalness of cow–calf separation is a continued cause for public criticism. This information provides further insight into citizens' concerns around cow–calf separation, which must be addressed to maintain the dairy industry's social license to operate. Although factorial surveys are beneficial in that they closely align with real-life decision-making, further qualitative exploration of the findings in this study is required to better understand the reasoning behind respondent decisions.

## Data Availability

The raw data supporting the conclusions of this article will be made available by the authors, without undue reservation.
