# Supplementary material for: Changing the frame: the impact of welfare narrative presentation on citizen choices about cow–calf separation
Source: Front Vet Sci. 2026 Sep 14;13:1813343. doi: 10.3389/fvets.2026.1813343 (PMC13617064; doi:10.3389/fvets.2026.1813343)
Supplement: Supplementary file 1 [file Table_1.docx]

Supplementary Material

# 1 Supplementary File 1. A copy of the survey distributed to the respondents

**Introduction**

Thank you for taking part in our survey. Your time and interest is very important to the success of this study. Your answers to this survey, which will remain anonymous, will allow us to better understand the public's perception of cow-calf separation and its influence on calf welfare.

The survey will begin with a few questions about you and your background.

**Section A: About you**

1. **What gender do you identify as?**

☐ Male	☐ Female	☐ Other	☐ Prefer not to say

2. **What age group do you belong to?**

☐ 18-24 ☐ 25-34 ☐ 35-44 ☐ 45-54 ☐ 55-64 ☐ 65 and over

3. **In which region do you currently live?**

☐ NE England ☐ NW England ☐ Yorkshire and the Humber ☐ East Midlands ☐ West Midlands ☐ East England ☐ London ☐ SE England ☐ SW England ☐ N Ireland ☐ Scotland ☐ Wales

4. **What is the highest level of education you have achieved?**

☐ None ☐ School ☐ College diploma/A-Levels ☐ Undergraduate degree ☐ Postgraduate degree ☐ Vocational/Skills-based

5. **How would you describe your knowledge about the dairy industry and how it operates?**

☐ I do not know anything about dairy farming

☐ I know a little bit about dairy farming because of social media and/or mainstream news

☐ I know a little bit about dairy farming because I read agricultural articles, attend open farms, or attend livestock/dairy events

☐ I know a lot about dairy farming by possessing a qualification relevant to agriculture, working with dairy cattle myself, or having family links to farming

6. **What links or experience do you have with the dairy industry? Please tick all that apply.**

☐ No links or experiences

☐ Visited a working dairy farm a few times

☐ Have friends or non-immediate family that farm or are involved with farming

☐ Have a job that involves working with farmers and/or the dairy industry

☐ Have immediate family involved with farming and/or I farm myself

7. **Which of these best describes your dietary preference?**

☐ **Vegetarian**: A plant-based diet avoiding meat, poultry and fish

☐ **Vegan**: A plant-based diet, avoiding all animal and animal-based products

☐ **Dairy-free**: A diet avoiding all dairy products such as cheese, milk, yoghurt and butter

☐ **Pescetarian**: Mainly a plant-based diet, with the inclusion of fish but not meat

☐ **Omnivore**: A diet that incorporates most foods including meat, fish and dairy

☐ **Flexitarian**: Mainly a plant-based diet, with the occasional inclusion of meat, fish and dairy

☐ Other, please specify:

8. **What is your main source of information about the dairy industry?**

☐ Social media

☐ Mainstream news or television programmes

☐ Agricultural magazines or newsletters

☐ Friends or family that are farmers

☐ Friends or family that work with farmers or the dairy industry in some capacity

☐ Scholarly articles

☐ Your job (if working in the agricultural sector)

☐ Other, please specify:

9. **Which of these best describes the type of areas you’ve lived in for most of your life?**

☐ Urban: Towns or cities

☐ Suburban: Areas just outside of towns or cities

☐ Rural: In the countryside or an area with lots of open land

☐ A mix of areas, none of which have been rural

☐ A mix of areas, including some rural living

☐ Other, please specify:

10. **On average, what is your household take-home income annually?**

☐ £20,000 or less

☐ £20,001-£30,000

☐ £30,001-£40,000

☐ £40,001-£50,000

☐ £50,001-£60,000

☐ £60,001-£70,000

☐ £70,001+

☐ Prefer not to say

Thank you for completing that section. We are now going to move on to the questions about cow-calf separation.

**Section B: Scenarios**

It is common practice on dairy farms in the United Kingdom to separate newborn calves from the cow within 24-48 hours of birth, although some farms may allow for a longer period of contact.

1. **Were you aware that this practice occurred on dairy farms in the United Kingdom?**

☐ Yes ☐ No

2. **To what extent do you support or oppose this practice?**

☐ Strongly support ☐ Somewhat support ☐ Neither support nor oppose ☐ Somewhat oppose ☐ Strongly oppose

A dairy farmer has recently implemented a new management practice on their farm, whereby calves are now separated from the cow within 24 hours of birth. The farmer has been monitoring the calves to determine the effect this early separation has had on their subsequent welfare.

You will now be presented with eight different scenarios that give insight into the calf's life in the days following separation from the cow. Changes in each scenario are shown by ***bold, italic font***. You will be asked to rate the extent to which you agree with the separation in the scenario, and also the extent to which you believe the calf's welfare has been affected.

**Scenario 1**

For one of the calves being monitored, the following findings have been observed. The calf has ***consumed an adequate amount of good quality colostrum, administered by the farmer, which is important for establishing early-life immunity*.** In the housing pen the calf has ***been avoiding novel objects in the pen***.

In recent health inspections, the calf has ***been recorded as having a below average daily weight gain***. Over the past few days, the calf has been ***demonstrating abnormal behaviours such as tongue rolling and sucking on pen fixtures***. Finally, the calf has exhibited **s*igns of insecurity due to the lack of maternal bonding***.

1. **Based only on the information above, to what extent do you agree with the farmer's choice to separate the calf from the cow given the outcome in this case?**

| Definitely do not support | | |  |  | Not sure |  |  | Definitely do  support | | |
| --- | --- | --- | --- | --- | --- | --- | --- | --- | --- | --- |
| -5 | **-4** | **-3** | **-2** | **-1** | **0** | **1** | **2** | **3** | **4** | **5** |

2. **To what extent do you believe the calf's welfare has been affected by separation from the cow in this case?**

| Welfare has  been negatively affected | | |  |  | Welfare has not been affected |  |  | Welfare has  been  positively  affected | | |
| --- | --- | --- | --- | --- | --- | --- | --- | --- | --- | --- |
| -5 | **-4** | **-3** | **-2** | **-1** | **0** | **1** | **2** | **3** | **4** | **5** |

**Scenario 2**

For one of the calves being monitored, the following findings have been observed. The calf has ***consumed an adequate amount of good quality colostrum, administered by the farmer, which is important for establishing early-life immunity***. In the housing pen the calf has ***greater space availability due to the absence of the cow***.

In recent health inspections, the calf has ***been recorded as having a below average daily weight gain***. Over the past few days, the calf has been ***demonstrating abnormal behaviours such as tongue rolling and sucking on pen fixtures***. Finally, the calf has exhibited ***no signs of separation stress***.

1. **Based only on the information above, to what extent do you agree with the farmer's choice to separate the calf from the cow given the outcome in this case?**

| Definitely do not support | | |  |  | Not sure |  |  | Definitely do  support | | |
| --- | --- | --- | --- | --- | --- | --- | --- | --- | --- | --- |
| -5 | **-4** | **-3** | **-2** | **-1** | **0** | **1** | **2** | **3** | **4** | **5** |

2. **To what extent do you believe the calf's welfare has been affected by separation from the cow in this case?**

| Welfare has  been negatively affected | | |  |  | Welfare has not been affected |  |  | Welfare has  been  positively  affected | | |
| --- | --- | --- | --- | --- | --- | --- | --- | --- | --- | --- |
| -5 | **-4** | **-3** | **-2** | **-1** | **0** | **1** | **2** | **3** | **4** | **5** |

**Scenario 3**

For one of the calves being monitored, the following findings have been observed. The calf has ***consumed an adequate amount of good quality colostrum, administered by the farmer, which is important for establishing early-life immunity***. In the housing pen the calf has ***greater space availability due to the absence of the cow***.

In recent health inspections, the calf has ***been recorded as having a below average daily weight gain***. Over the past few days, the calf has been ***socially interacting with other calves in the pen, forming friendship bonds***. Finally, the calf has exhibited ***signs of insecurity due to the lack of maternal bonding***.

1. **Based only on the information above, to what extent do you agree with the farmer's choice to separate the calf from the cow given the outcome in this case?**

| Definitely do not support | | |  |  | Not sure |  |  | Definitely do  support | | |
| --- | --- | --- | --- | --- | --- | --- | --- | --- | --- | --- |
| -5 | **-4** | **-3** | **-2** | **-1** | **0** | **1** | **2** | **3** | **4** | **5** |

2. **To what extent do you believe the calf's welfare has been affected by separation from the cow in this case?**

| Welfare has  been negatively affected | | |  |  | Welfare has not been affected |  |  | Welfare has  been  positively  affected | | |
| --- | --- | --- | --- | --- | --- | --- | --- | --- | --- | --- |
| -5 | **-4** | **-3** | **-2** | **-1** | **0** | **1** | **2** | **3** | **4** | **5** |

**Scenario 4**

For one of the calves being monitored, the following findings have been observed. The calf has ***consumed an adequate amount of good quality colostrum, administered by the farmer, which is important for establishing early-life immunity***. In the housing pen the calf has ***greater space availability due to the absence of the cow***.

In recent health inspections, the calf has ***not been diagnosed with Failure of Passive Transfer, a condition causing lifelong susceptibility to diseases***. Over the past few days, the calf has been ***socially interacting with other calves in the pen, forming friendship bonds***. Finally, the calf has exhibited ***no signs of separation stress***.

1. **Based only on the information above, to what extent do you agree with the farmer's choice to separate the calf from the cow given the outcome in this case?**

| Definitely do not support | | |  |  | Not sure |  |  | Definitely do  support | | |
| --- | --- | --- | --- | --- | --- | --- | --- | --- | --- | --- |
| -5 | **-4** | **-3** | **-2** | **-1** | **0** | **1** | **2** | **3** | **4** | **5** |

2. **To what extent do you believe the calf's welfare has been affected by separation from the cow in this case?**

| Welfare has  been negatively affected | | |  |  | Welfare has not been affected |  |  | Welfare has  been  positively  affected | | |
| --- | --- | --- | --- | --- | --- | --- | --- | --- | --- | --- |
| -5 | **-4** | **-3** | **-2** | **-1** | **0** | **1** | **2** | **3** | **4** | **5** |

**Scenario 5**

For one of the calves being monitored, the following findings have been observed. The calf has ***a low daily milk intake due to smaller volumes being available***. In the housing pen the calf has ***greater space availability due to the absence of the cow.***

In recent health inspections, the calf has ***been recorded as having a below average daily weight gain***. Over the past few days, the calf has been ***socially interacting with other calves in the pen, forming friendship bonds***. Finally, the calf has exhibited ***no signs of separation stress***.

1. **Based only on the information above, to what extent do you agree with the farmer's choice to separate the calf from the cow given the outcome in this case?**

| Definitely do not support | | |  |  | Not sure |  |  | Definitely do  support | | |
| --- | --- | --- | --- | --- | --- | --- | --- | --- | --- | --- |
| -5 | **-4** | **-3** | **-2** | **-1** | **0** | **1** | **2** | **3** | **4** | **5** |

2. **To what extent do you believe the calf's welfare has been affected by separation from the cow in this case?**

| Welfare has  been negatively affected | | |  |  | Welfare has not been affected |  |  | Welfare has  been  positively  affected | | |
| --- | --- | --- | --- | --- | --- | --- | --- | --- | --- | --- |
| -5 | **-4** | **-3** | **-2** | **-1** | **0** | **1** | **2** | **3** | **4** | **5** |

**Scenario 6**

For one of the calves being monitored, the following findings have been observed. The calf ***a low daily milk intake due to smaller volumes being available***. In the housing pen the calf has ***been avoiding novel objects in the pen***.

In recent health inspections, the calf has ***not been diagnosed with Failure of Passive Transfer, a condition causing lifelong susceptibility to diseases***. Over the past few days, the calf has been ***socially interacting with other calves in the pen, forming friendship bonds***. Finally, the calf has exhibited ***no signs of separation stress***.

1. **Based only on the information above, to what extent do you agree with the farmer's choice to separate the calf from the cow given the outcome in this case?**

| Definitely do not support | | |  |  | Not sure |  |  | Definitely do  support | | |
| --- | --- | --- | --- | --- | --- | --- | --- | --- | --- | --- |
| -5 | **-4** | **-3** | **-2** | **-1** | **0** | **1** | **2** | **3** | **4** | **5** |

2. **To what extent do you believe the calf's welfare has been affected by separation from the cow in this case?**

| Welfare has  been negatively affected | | |  |  | Welfare has not been affected |  |  | Welfare has  been  positively  affected | | |
| --- | --- | --- | --- | --- | --- | --- | --- | --- | --- | --- |
| -5 | **-4** | **-3** | **-2** | **-1** | **0** | **1** | **2** | **3** | **4** | **5** |

**Scenario 7**

For one of the calves being monitored, the following findings have been observed. The calf has ***a low daily milk intake due to smaller volumes being available***. In the housing pen the calf has ***been avoiding novel objects in the pen***.

In recent health inspections, the calf has ***been recorded as having a below average daily weight gain***. Over the past few days, the calf has been ***demonstrating abnormal behaviours such as tongue rolling and sucking on pen fixtures***. Finally, the calf has exhibited ***no signs of separation stress***.

1. **Based only on the information above, to what extent do you agree with the farmer's choice to separate the calf from the cow given the outcome in this case?**

| Definitely do not support | | |  |  | Not sure |  |  | Definitely do  support | | |
| --- | --- | --- | --- | --- | --- | --- | --- | --- | --- | --- |
| -5 | **-4** | **-3** | **-2** | **-1** | **0** | **1** | **2** | **3** | **4** | **5** |

2. **To what extent do you believe the calf's welfare has been affected by separation from the cow in this case?**

| Welfare has  been negatively affected | | |  |  | Welfare has not been affected |  |  | Welfare has  been  positively  affected | | |
| --- | --- | --- | --- | --- | --- | --- | --- | --- | --- | --- |
| -5 | **-4** | **-3** | **-2** | **-1** | **0** | **1** | **2** | **3** | **4** | **5** |

**Scenario 8**

For one of the calves being monitored, the following findings have been observed. The calf has ***consumed an adequate amount of good quality colostrum, administered by the farmer, which is important for establishing early-life immunity***. In the housing pen the calf has ***been avoiding novel objects in the pen***.

In recent health inspections, the calf has ***not been diagnosed with Failure of Passive Transfer, a condition causing lifelong susceptibility to diseases***. Over the past few days, the calf has been ***demonstrating abnormal behaviours such as tongue rolling and sucking on pen fixtures***. Finally, the calf has exhibited ***no signs of separation stress***.

1. **Based only on the information above, to what extent do you agree with the farmer's choice to separate the calf from the cow given the outcome in this case?**

| Definitely do not support | | |  |  | Not sure |  |  | Definitely do  support | | |
| --- | --- | --- | --- | --- | --- | --- | --- | --- | --- | --- |
| -5 | **-4** | **-3** | **-2** | **-1** | **0** | **1** | **2** | **3** | **4** | **5** |

2. **To what extent do you believe the calf's welfare has been affected by separation from the cow in this case?**

| Welfare has  been negatively affected | | |  |  | Welfare has not been affected |  |  | Welfare has  been  positively  affected | | |
| --- | --- | --- | --- | --- | --- | --- | --- | --- | --- | --- |
| -5 | **-4** | **-3** | **-2** | **-1** | **0** | **1** | **2** | **3** | **4** | **5** |

# 2 Supplementary File 2. The sociodemographic characteristics of the survey respondents (n = 503).

| **Variable** | **Number** | **Percentage (%)** |
| --- | --- | --- |
| **Gender**  Male  Female  Other  Prefer not to say | 247  252  3  1 | 49.11  50.10  0.60  0.20 |
| **Age**  18-24  25-34  35-44  45-54  55-64  65 and over | 49  90  84  79  82  119 | 9.74  17.89  16.70  15.71  16.30  23.66 |
| **Region**  NE England  NW England  Yorkshire and the Humber  East Midlands  West Midlands  East England  London  SE England  SW England  N Ireland  Scotland  Wales | 23  56  43  36  44  45  67  73  40  14  40  22 | 4.57  11.13  8.55  7.16  8.75  8.95  13.32  14.51  7.95  2.78  7.95  4.37 |
| **Level of Education**  None  School  College diploma/A-levels  Undergraduate degree  Postgraduate degree  Vocational/Skills-based | 18  129  127  101  101  27 | 3.58  25.65  25.25  20.08  20.08  5.37 |
| **Knowledge of Dairy Industry**  I do not know anything about dairy farming  I know a little bit about dairy farming because of social media and/or mainstream news  I know a little bit about dairy farming because I read agricultural articles, attend open farms, or attend livestock/dairy events  I know a lot about dairy farming by possessing a qualification relevant to agriculture, working with dairy cattle myself, or having family links to farming | 154  209  100  40 | 30.62  41.55  19.88  7.95 |
| **Experience with Dairy Industry**  No links or experiences  Visited a working dairy farm a few times  Have friends or non-immediate family that farm or are involved with farming  Have a job that involves working with farmers and/or the dairy industry  Have immediate family involved with farming and/or I farm myself | 287  113  97  26  28 | 57.06  22.47  19.28  5.17  5.57 |
| **Dietary Preference**  Vegetarian  Vegan  Dairy-free  Pescetarian  Omnivore  Flexitarian  Other | 39  16  42  26  311  66  3 | 7.75  3.18  8.35  5.17  61.83  13.12  0.60 |
| **Source of Information about Dairy Industry**  Social media  Mainstream news or television programmes  Agricultural magazines or newsletters  Friends or family that are farmers  Friends or family that work with farmers or the dairy industry in some capacity  Scholarly articles  Your job (if working in the agricultural sector)  Other | 146  200  27  41  47  9  4  29 | 29.03  39.76  5.37  8.15  9.34  1.79  0.80  5.77 |
| **Types of Areas Lived**  Urban  Suburban  Rural  Mixture, none of which are rural  Mixture, with some rural living  Other | 216  170  58  25  32  2 | 42.94  33.80  11.53  4.97  6.36  0.40 |
| **Household Income**  £20,000 or less  £20,001-£30,000  £30,001-£40,000  £40,001-£50,000  £50,001-£60,000  £60,001-£70,000  £70,001 or over  Prefer not to say | 124  85  68  62  41  19  76  28 | 24.65  16.90  13.52  12.33  8.15  3.78  15.11  5.57 |
